# Influence of Membrane Vibration on Particles Rejection Using a Slotted Pore Membrane Microfiltration

**DOI:** 10.3390/membranes11090709

**Published:** 2021-09-15

**Authors:** Asmat Ullah, Kamran Alam, Saad Ullah Khan, Victor M. Starov

**Affiliations:** 1 Department of Chemical Engineering, University of Engineering and Technology Peshawar, Peshawar 25000, Pakistan; 2 Faculty of Materials and Chemical Engineering, GIK Institute of Engineering Sciences and Technology, Topi 23460, Pakistan; kalam3@asu.edu (K.A.); saadkhan7907@gmail.com (S.U.K.); 3 Department of Chemical Engineering, Loughborough University, Loughborough LE11 3TU, UK

**Keywords:** membrane oscillation, shear rate, slotted structure membrane, oil water separation, membrane fouling

## Abstract

A new method is proposed to increase the rejection in microfiltration by applying membrane oscillation, using a new type of microfiltration membrane with slotted pores. The oscillations applied to the membrane surface result in reduced membrane fouling and increased separation efficiency. An exact mathematical solution of the flow in the surrounding solution outside the oscillating membrane is developed. The oscillation results in the appearance of a lift velocity, which moves oil particles away from the membrane. The latter results in both reduced membrane fouling and increased oil droplet rejection. This developed model was supported by the experimental results for oil water separation in the produced water treatment. It was proven that the oil droplet concentration was reduced notably in the permeate, due to the membrane oscillation, and that the applied shear rate caused by the membrane oscillation also reduced pore blockage. A four-times lower oil concentration was recorded in the permeate when the membrane vibration frequency was 25 Hz, compared to without membrane vibration. Newly generated microfiltration membranes with slotted pores were used in the experiments.

## 1. Introduction

Sea water is substantially polluted by the discharge of produced oily water, which is an important environmental concern [1,2,3]. On an industrial scale, oily water is generated in huge amounts, with predictions of approximately 88 billion barrels on a yearly basis worldwide [4,5]. There are various separation methods used for purification of the produced oily water, which include pH change, gravitational methods, centrifugation, biological treatment, membrane filtration, and electrostatic de-emulsification [6,7]. The membrane purification process is superior to the other processes because it has several benefits, such as requiring no chemicals [8,9], low input energy [10], environment friendliness [11,12], and a high quality of permeate [13]. This is the reason why membrane separation processes have gained the attention of researchers over recent years for the treatment of produced water [14,15]. Despite the wide application of membrane purification, fouling is a major problem in the membrane separation of oil from water in emulsions and leads to a considerable reduction in the permeate flux [16,17,18]. 

Various methods of membrane separation have been tried for the separation of oil droplets and water [17,18,19]. The methods of microfiltration (MF) [20,21,22,23,24,25,26] and ultrafiltration (UF) [27,28,29,30,31,32] are considered superior compared to other membrane separation methods for separation of oil from water. The reason being that the fouling in these membranes is less than that of reverse osmosis and nano-filtration membranes for oil–water separation. It has been found that a MF membrane gives greater permeate flux at lower trans membrane pressures compared to UF processes. MF is more economical for oil water separation at a commercial level [33,34,35]. Many researchers have also studied the impact of membrane pore geometry on separation efficiency [36,37,38,39]. Recently, slotted pore membranes were introduced for oil–water emulsion purification [24,35,40,41,42,43,44]. It was found that a slotted pore membrane gives a greater value of permeate flux at a lower transmembrane pressure compared to circular pore membranes [35]. 

MF is widely used; however, membrane fouling still remains an important issue to be resolved [44]. Various methods have been suggested for the reduction of membrane fouling of increasing shear, such as higher cross flow filtration and aeration. In the aeration process, bubbles are generated which results in disturbing the concentration polarization and, hence, reduces the fouling [45]. However, this process has the disadvantage of higher energy consumption. In the case of cross-flow filtration, the feed is supplied with a high tangential velocity, which results in shear rate generation over the membrane surface [45], but this approach also has the limitation of high power consumption and requires multiple recirculations of the feed stream [45]. To overcome this problem of higher energy consumption in cross flow filtration, another method, referred to as dynamic microfiltration, was proposed. In dynamic microfiltration, a relative motion between the bulk fluid and membrane used is applied, which results in a higher shear rate applied to the membrane surface. This is achieved by vibration or rotation of the membranes. With the application of membrane oscillation, fouling and concentration polarization can be reduced [40], and just this method is considered below. It is shown below that membrane oscillation results in a substantial decrease of the transmembrane pressure, results in a higher rejection of oil droplets, and substantially decreases membrane fouling. It is shown below that the membrane oscillation method provides lower oil concentrations in the permeate; that is, a higher rejection compared with other methods. 

A mathematical model is developed below and the exact solution of the flow in the vicinity of the oscillating membrane is deduced. The deduced mathematical solution allows calculating the shear applied on the membrane surface. It is shown that the imposed oscillations allow reduced membrane fouling and increased rejection of oil droplets, which is caused by the lift velocity. It is shown that, because of the oscillations that are applied over the membrane’s surface, lift velocities are developed, which move the droplets from the membrane’s surface. The effect of membrane pore blocking was studied at various shear rates. An experimental investigation of the oscillating membrane fouling was undertaken, which was in agreement with the theoretical prediction. A prediction of the permeate concentration of oil droplets was made. 

In the recent years, the application of membrane technology for oil–water separation has been investigated by many researches [46,47,48]. Generally, the efficient separation of oil from water has been the main theme of research [47]. However, membrane fouling in oil–water separation is huge problem and little attention has been given to it. In the current study, the prime focus is how membrane oscillation reduces fouling; for that reason a new type of membrane with slotted pores was used for investigation of the influence of oscillation, and which have not been used for this purpose before. 

## 2. Mathematical Model

**Figure 1:**
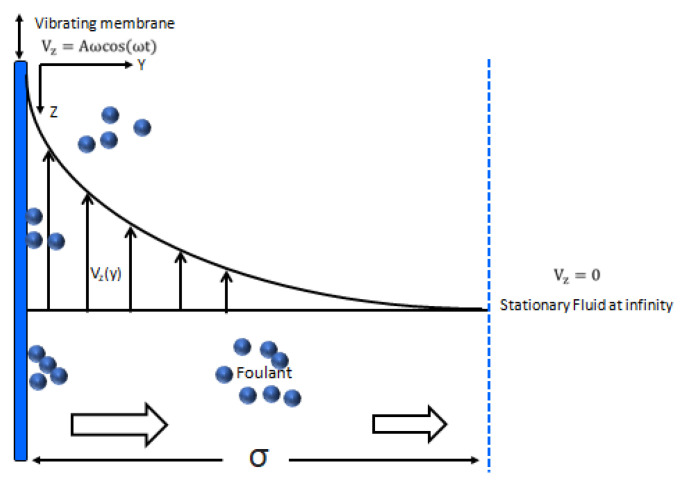
Velocity distribution of the fluid and motion of foulants in the vicinity of the submerged oscillating membrane.

In Figure 1 a schematic diagram of the membrane oscillating along the z-axis is presented. The velocity distribution was generated because of the oscillating membrane, which results in the creation of the shear rate. The oscillation results in the lift velocity of the oil droplets, which move away the fouling materials from surface of the membrane and reduce the concentration polarization in the vicinity of the membrane. As a result, both the fouling and concentration polarization are reduced. The model developed in this section allows calculating the shear rate over the membrane’s surface caused by membrane oscillations. The dimension of the membrane module is supposed to be much bigger compared with the dimension, δ, of the region caused by the oscillations (Figure 1); that is, the membrane can be assumed to be flat. The oscillating membrane is completely immersed in the liquid. It is also assumed that the Reynolds number is small. In this case the Navier–Stokes equation is reduced.to the following:

(1) $$\frac{\partial V_{z}}{\partial t}=\nu \left(\frac{\partial^{2}V_{z}}{\partial y^{2}}\right)$$

where V_z_ is the z component of the fluid velocity, which depends on the y only (Figure 1), t and y are the time and co-ordinate (Figure 1), and ν is the kinematic liquid viscosity. The displacement of the membrane which is oscillating along the z-axis is as follows (Figure 1):

Z(t)=A sin(ωt)

where A is an amplitude of oscillations and ω is the frequency of oscillations.

The following boundary conditions should be satisfied for the liquid velocity, determined by Equation (1):

(2) $$V_{z}=A\omega \cos(\omega t),\;y=0,$$

(3) $$\mathrm{At}\;y\to \infty {\;V}_{z}\to 0,\;y\to \infty .$$

The exact solution of Equation (1) satisfying boundary conditions (2) and (3) is as follows:

(4) $$V_{z}=A\omega \exp\left(-\alpha y\right)\cos\left(\alpha y-\omega t\right),$$

where $\alpha =\sqrt{\omega /2\nu }$. See Appendix for details of the derivation. 

According to Equation (4), the influence of oscillations is extended into the bulk of the liquid on the distance $\delta \sim \frac{1}{\alpha }=\sqrt{2\nu /\omega }$, that is, it decreases with increasing frequency of the oscillations. 

We define the shear rate as $\gamma {\left(t,y\right)}_{}=\frac{{\mathrm{dV}}_{z}\left(t,y\right)}{\mathrm{dy}}$. Using Equation (4) we conclude:

(5) $$\gamma {\left(t,y\right)}_{}=\frac{{\mathrm{dV}}_{z}\left(t,y\right)}{\mathrm{dy}}=-A\omega \alpha \exp\left(-\alpha y\right)\left[\cos\left(\alpha y-\omega t\right)+\sin\left(\alpha y-\omega t\right)\right]$$

The shear rate over the surface of the oscillating membrane can be obtained by finding the value for the boundary at y = 0. This gives the shear rate over the surface of the oscillating membrane:

(6) $$\gamma_{s}=A\omega \alpha \left[\sin\left(\omega t\right)-\cos\left(\omega t\right)\right]$$

**Figure 2:**
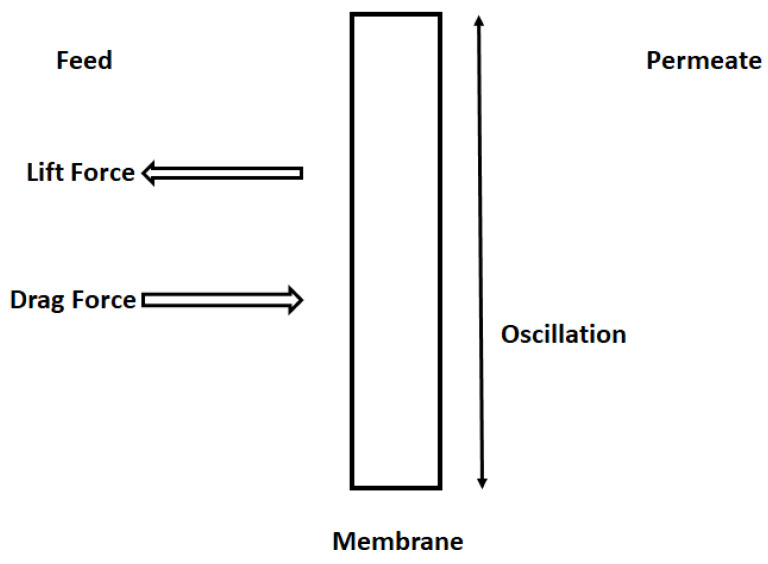
Drag force tries to push the drops to the permeate side, while the lift force coming from the membrane oscillation acts in the opposite direction to the drag force.

The shear applied to the membrane surface is proportional to the frequency of the oscillations according to Equation (6). This shear rate prevents pore blocking. The latter has a direct experimental confirmation (see figures in the results and discussion section), which proves that pore blocking is reduced linearly with oscillation frequency, in accordance with Equation (6). In the absence of the oscillations, the only force acting on the oil droplet is the drag force, which pushes the droplet through the membrane into the permeate. However, when the membrane is oscillating this results in the generation of the shear of various intensities and the lift force, which moves the oil droplet away from the membrane surface. Figure 2 shows the action of the forces (drag and lift forces) in the case of membrane oscillation. Lift force is the consequence of the oscillations applied to the membrane surface. The intensity of the lift force is higher at the membrane surface and gradually decreases when moving away from the membrane surface. 

Expressions for the drag force and lift force are given below:

(7) $$F_{d}=k_{w}12\pi \eta R_{sp}U$$

where $F_{d}$ is the drag force, $k_{w}$ is a wall correction factor (for a similar system a $k_{w}$ value of 4.3 was used [24]), η is the dynamic viscosity of the liquid, $R_{sp}$ is the radius of the droplet, and U is the permeate velocity of the liquid, caused by applied cross-membrane pressure difference [24]. 

The lift force is given by the following expression [49]:

(8) $$F_{l}=81.2\left({\left(\rho_{w}\eta {\left|\gamma \right|}^{3}\right)}^{0.5}\right)R_{sp}{}^{3}$$

where F_l_ is the lift force, $\rho_{w}$ is the density of the water at room temperature, and γ, according to Equation (6), is the applied shear rate [46]. Equation (8) shows that the lift force is determined by the applied shear rate and drop size. This means that for a given shear rate, the shear force will be more effective for larger droplets [49]. Notably, according to Equations (6) and (8), the lift force decreases exponentially away from the oscillation membrane surface.

The shear rate is produced over the surface of the membrane because oscillation creates the lift force which tends to move the droplets from the surface of the membrane. As a result, the number oil drops which could be deposited on the membrane surface and block membrane pores is reduced. Therefore, the fouling also gets reduced. The model is validated with the experimental results in the following section. A lower concentration of oil droplets in the permeate which is due to the shear rate applied over the membrane surface has been investigated.

According to Figure 2, the drag velocity of oil droplets towards the oscillating membrane is reduced (or even becomes negative; that is, away from the oscillating membrane), which is caused by the applied membrane oscillations. 

## 3. Experimental Methodology

### 3.1. Oscilating Membrane Filtration 

**Figure 3:**
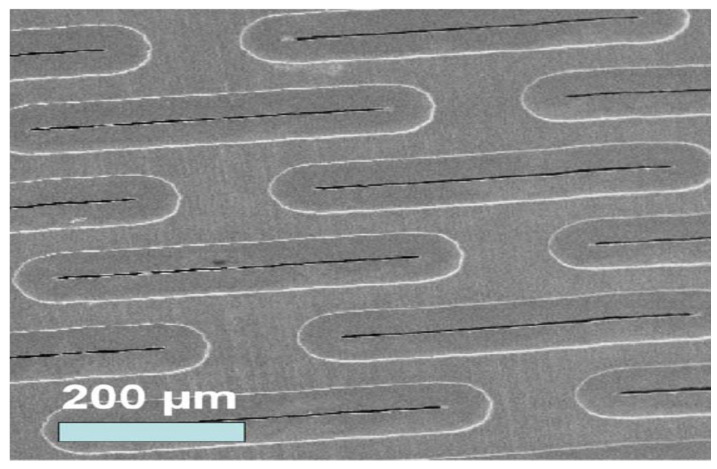
Scanning Electron Microscopy (SEM) Diagram of Slotted Porous Structure Membrane.

A food blender at high speed was used for the oil droplet formation from vegetable oil. Real produced water was also used in the experiments. A Coulter Mullisizer II was used for the examination of oil droplet size distribution in both cases. The size scale of oil droplets in both cases was determined to be in the range of 1 μm to 15 μm. A slotted porous membrane of 4 μm pore size in width was utilized for the process of oil and water separation (Figure 3). This membrane was made up of Nickel (Ni), whose surface was modified with Poly tetra fluoro ethylene (PTFE) by Micropore Technologies, Lazenby, UK. A picture of the membrane captured by scanning electron microscopy is shown in Figure 3. 

**Figure 4:**
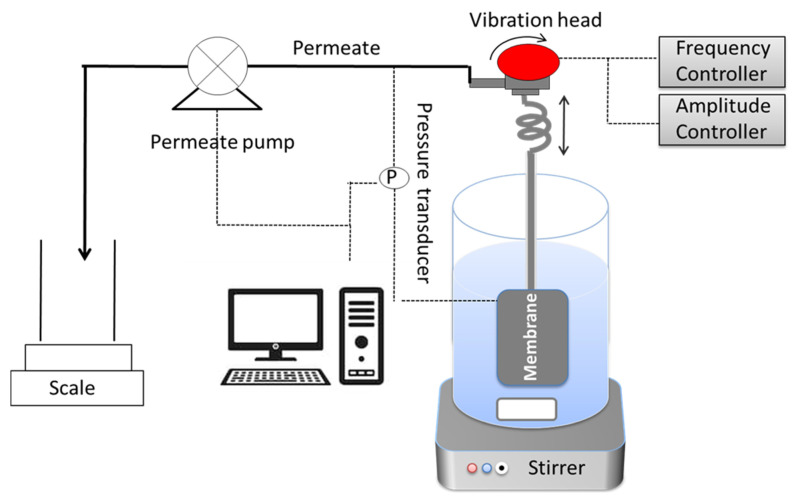
Flow diagram of the membrane oscillation devise.

The membrane was connected with an oscillating arm, then the oscillating arm was initiated with an electrochemical oscillator in order to create shear rate over the membrane surface. A flow diagram of the oscillating microfiltration membrane with oscillating system for separating oil and water is shown in Figure 4. The oscillating frequency and amplitude scale for membranes was varied between 0 and 10 Hz, and 0 and 10 mm, respectively. As a result, the shear rate was produced in the vicinity of the membrane outer surface. The constant flux through the membrane was created using a positive displacement (PD) pump. 

Filtration experiments with the oscillating slotted pore membrane at various intensities were applied to both crude oil and vegetable oil emulsions. The membrane oscillation in the vertical direction resulted in the shear rate creation. 

### 3.2. Materials Used 

Drops of crude oil and vegetable oil (stabilized by Tween 20) were used. In both cases oil droplets showed interfacial tension around 30 and 4 mn/m, accordingly. The length and width values of the slotted pores on the membrane surface were 400 μm and 4 μm, respectively. The total area of the slotted pore membrane was 1.6 × 10^−9^ m^2^.

## 4. Results and Discussion

**Figure 5:**
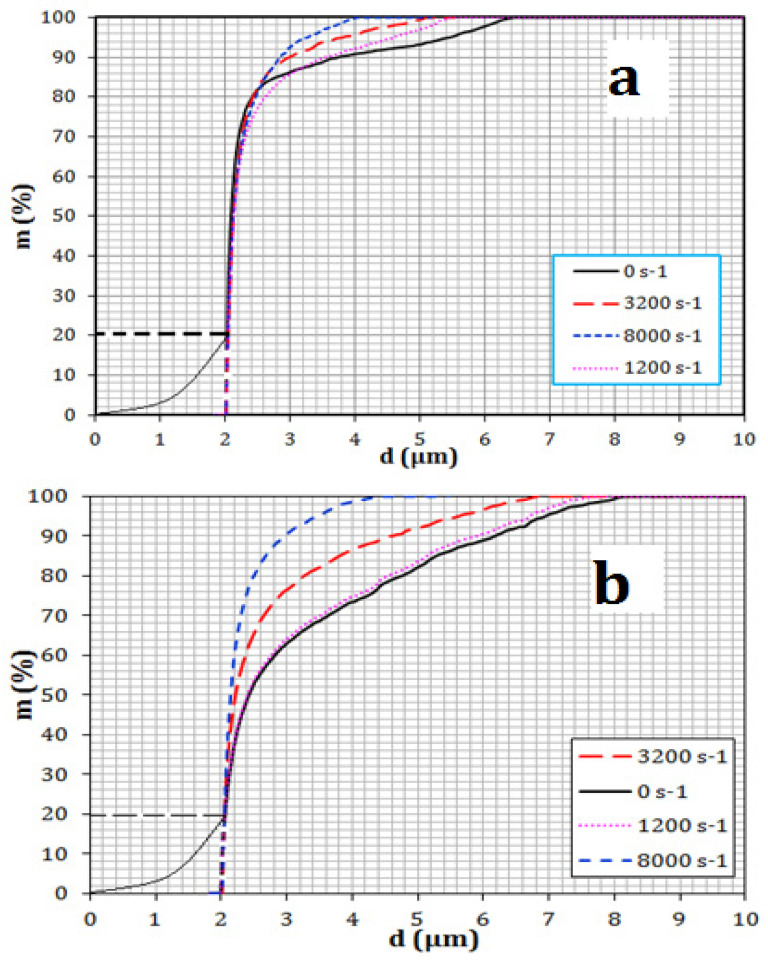
Size distribution of the mass of oil droplets stabilized by Tween 20 in the permeate at (**a**) at 200 lm^−2^h^−1^ and (**b**) 1000 lm^−2^h^−1^. [44].

**Figure 6:**
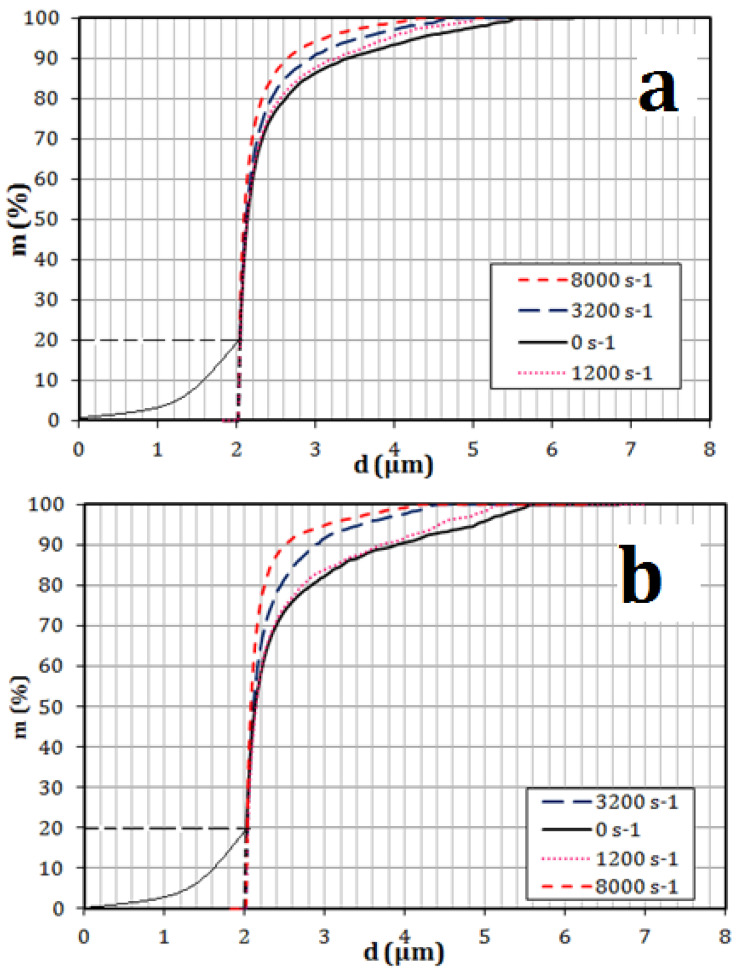
Size distribution of the mass of crude oil droplets in the permeate at (**a**) 200 lm^−2^h^−1^ (**b**) 1000 lm^−2^h^−1^ [44].

The size distribution was established based on the oil droplet mass in the permeate stream, as shown in Figure 5 and Figure 6. Figure 5 and Figure 6 show that there was a considerable reduction in the concentration of oil droplets in the permeate, caused by the membrane oscillations applied. The reduction in the concentration of permeate was found to be a linear function of the applied shear rate. The interfacial tension played an important part in reducing the oil droplet concentration. At low values of interfacial tension, flattening and deformation of oil droplets was easier, which tended to move droplets into the permeate. While at high values of interfacial tension, the droplets became stiffer, their deformation and flattening was not so easy, and this resulted in a lower droplet penetration into the permeate. 

**Figure 7:**
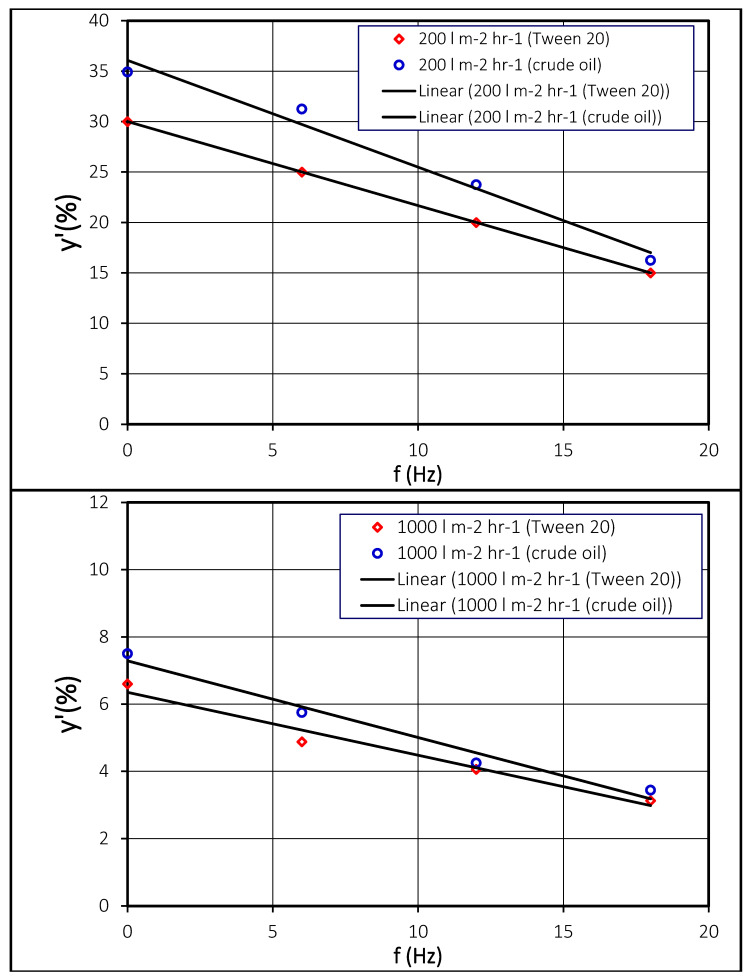
Impact of oscillation frequency, f(HZ), on the pore blockage area, y’, in percentage. Stable vegetable oil droplets (stabilized by Tween 20) and crude oil droplets at (**a**) 200 lm^−2^h^−1^ (**b**) 1000 lm^−2^h^−1^.Table 1 shows the number of crude oil drops in the permeate and permeate concentration obtained at different shear rates. It is clear that both the number of drops in the permeate and the crude oil concentration in the permeate were influenced by the applied shear. The number of drops and crude oil concentration were reduced with the applied shear rate.

The blocking of the pores (slots) of membrane was investigated experimentally by applying an oscillation shear rate over the membrane surface. In these experiments, droplets of crude oil and vegetable oil (stabilized by Tween 20) were used. Similarly to the concentration in permeate, it was found that the blockage of membrane pores was also reduced by the applied oscillating membrane. It was found that the blockage of pores was appreciably reduced by the oscillations applied, as shown in Figure 7. This reduction in blockage was found to be a linear function of the oscillating frequency of the membrane. This means the greater value of the oscillating frequency of the membrane, the lower the blockage of pores. It was shown that the interfacial tension was an essential parameter in pore blockage of the membrane. Oil droplets having a low interfacial tension can be deformed easily and tend to move into the permeate, which results in less pore blockage. However, on the other hand, at high interfacial tension, the blockage in pores of the membrane was found to be higher. This made the drops stiffer, and it became more difficult to push the undeformed drops through the pores, which resulted in more pore blockage [44]. 

## 5. Conclusions

An analytical solution has been developed which allows calculating the velocity and shear rate distribution in the vicinity of the oscillating membrane and on the membrane surface itself. It was shown that the oscillations result in the creation of a lift force, which acts in the opposite direction compared with drag force. This results in a lower number of oil droplets reaching the oscillating membrane, and hence, less oil droplets penetrating into the permeate (higher rejections) and less membrane fouling. The analytical study was validated against the experimental results, which proved that the oscillation of the membrane reduced the concentration of oil droplets in the permeate and reduced the membrane fouling. This reduction in the oil droplet concentration in the permeate was found to be a linear function of the oscillating frequency of the membrane. The oscillations reduced the blockage of pores; the greater the shear rate intensity, the less pore blockage was noticed. 

## Figures and Tables

**Table 1:** Number of crude oil drops (30° American Petroleum Institute) and concentration of oil in the feed and permeate obtained at various frequencies of oscillation and a 400 l m^−2^ h^−1^ flux rate.

Frequency (Hz)	No of Drops per 0.4 mL Sample	Concentration of Crude Oil in the Feed (ppm)	Concentration of Crude Oil in the Permeate (ppm)
25	1020	400	5
21	1606	400	7
0	5092	400	21

## Nomenclature

A	Amplitude of oscillation (m)
Z(y,t)	Instantaneous displacement of an immersed oscillating membrane along z-axis (m)
t	Time (s)
V_z_	Velocity of the fluid in z direction (m/s)
y	Lateral coordinates (m)
z	Directional Coordinate (m)
Greek Symbols	
γ	Shear rate (1/s)
γ_s_	Shear rate at membrane surface (1/s)
µ	Fluid dynamic viscosity (kg/ms)
ʋ	Fluid kinematic viscosity (m^2^/s)
ρ	Fluid density (kg/m^3^)
ω	Angular Frequency (rad/s)
I	$\sqrt{-1}$
ℜe	Real part

## Appendix A

The solution of Equation (1) is obtained using (a) a method of separation variables and (b) the solution is tried in complex variable form, but in the end the real part of the deduced solution satisfies the initial equation and boundary conditions (2) and (3).

The solution of Equation (1) is presented in the following form

(A1) $$V_{z}=ℜef_{1}\left(t\right)f_{2}\left(y\right)$$

Substitution into Equation (1) and separating variable results in:

$$\frac{f_{1}^{\prime }\left(t\right)}{f_{1}\left(t\right)}=\nu f_{2}^{\prime \prime }\left(y\right)/f_{2}\left(y\right)$$

The left-hand site of the latter equation depends on time only, while the right-hand site on y only. The latter is possible only if both sides are constants. Bearing in mind that both sides should be oscillatory functions of ω, this constant is equal to iω, where i is the imaginary unit. The latter results in the following two equations

$$\frac{f_{1}^{\prime }\left(t\right)}{f_{1}\left(t\right)}=i\omega \;\frac{\nu f_{2}^{\prime \prime }\left(y\right)}{f_{2}\left(y\right)}=i\omega$$

The solution of these equations is as follows

(A2) $$f_{1}\left(t\right)=e^{i\omega }=cos\omega t+i\;sin\omega t$$

(A3) $$f_{2}\left(y\right)=A_{1}e^{\left(\alpha +i\alpha \right)y}+A_{2}e^{\left(-\alpha -i\alpha \right)y}=A_{1}e^{\alpha y}\left(\cos\alpha y+i\sin\alpha y\right)+A_{2}e^{-\alpha y}\left(\cos\alpha y-i\sin\alpha y\right)$$

where A_1_ and A_2_ are integration constants. From the condition (3) we can conclude that A_1_ = 0, otherwise the solution does not tend to zero, as *y* tends to infinity. From the condition (2) we conclude that $A_{2}=A\omega$. Hence,

(A4) $$f_{2}\left(y\right)=A\omega e^{-\alpha y}\left(\cos\alpha y-\mathrm{isin}\alpha y\right)$$

Substitution of expressions (A2) and (A4) into Equation (A1) results in the solution given by Equation (4).
